# Water-Content-Dependent Switching of the Bending Behavior of Photoresponsive Hydrogels Composed of Hydrophilic Acrylamide-Based Main Chains and Hydrophobic Azobenzene

**DOI:** 10.3390/gels9080658

**Published:** 2023-08-16

**Authors:** Junsu Park, Yuki Shimizu, Xin Zhou, Ryohei Ikura, Go Matsuba, Yoshinori Takashima

**Affiliations:** 1 Department of Macromolecular Science, Graduate School of Science, Osaka University, 1-1 Machikaneyama, Toyonaka 560-0043, Osaka, Japanikurar16@chem.sci.osaka-u.ac.jp (R.I.); 2 Forefront Research Center, Graduate School of Science, Osaka University, 1-1 Machikaneyama, Toyonaka 560-0043, Osaka, Japan; 3 Graduate School of Organic Materials Engineering, Yamagata University, 4-3-16 Jonan, Yonezawa 992-8510, Yamagata, Japan; gmatsuba@yz.yamagata-u.ac.jp; 4 Innovative Catalysis Science Division, Institute for Open and Transdisciplinary Research Initiatives (OTRI), Osaka University, 1-1 Yamadaoka, Suita 565-0871, Osaka, Japan; 5 Institute for Advanced Co-Creation Studies, Osaka University, 1-1 Yamadaoka, Suita 565-0871, Osaka, Japan

**Keywords:** photoresponsiveness, moisture sensitivity, bending behavior switching, phase separated structure, azobenzene

## Abstract

Photoresponsiveness is a promising characteristic of stimulus-responsive materials. Photoresponsiveness can be achieved by incorporating photoresponsive molecules into polymeric materials. In addition, multiple-stimuli-responsive materials have attracted scientists’ interest. Among the numerous multiple-stimuli-responsive materials, moisture- and photoresponsive materials are the focus of this report. These stimuli-responsive materials responded to the stimuli synergistically or orthogonally. Unlike most stimulus-responsive materials utilizing moisture and light as stimuli, the materials studied herein switch their photoresponsiveness in the presence of moisture. Appropriate copolymers consisting of hydrophilic acrylamide-based monomers for the main chain and hydrophobic azobenzene moieties switched their bending behaviors at 6–9 wt% water contents. At water contents lower than 6 wt%, the polymeric materials bent away from the light source, while they bent toward the light source at water contents higher than 10 wt%. At a low water content, the bending behaviors can be described on the molecular scale. At a high water content, the bending behavior requires consideration of the phase scale, not only the molecular scale. By controlling the balance between hydrophilicity and hydrophobicity, the switching behavior was achieved. This switching behavior may inspire additional strategies for the application of polymeric material as actuators.

## 1. Introduction

Numerous types of materials are utilized our society. For practical use, all materials must have environmental (or weather) resistivity unless they will be used in unique applications. Moisture and ultraviolet (UV) light are the major factors influencing the lifetime of materials. For example, polymers, regardless of their hydrophilicities, swell or hydrate with water when stored in humid environments [1,2,3,4,5]. Moreover, the water content (*W*_c_) significantly affects the mechanical and structural properties of hydrophilic polymers, particularly hydrogels [6,7,8,9,10,11,12,13]. As UV light has high energy, accumulated exposure to highly intense UV light can damage chemical bonds in polymeric materials [14,15,16,17]. While many materials have been designed considering their environmental resistivities, environmental factors have been utilized as stimuli to switch or control the properties or structures of some materials to prepare stimulus-responsive materials. Namely, when some materials change their structures or properties in response to water or light, they are water-responsive or photoresponsive materials, respectively.

Light is an attractive and easily accessible stimulus because light irradiation can be performed from a distant location, at a specific site (local irradiation), and with easy tunability of the wavelength and intensity. Actuating materials and self-healing materials are representative materials that use light as a stimulus. Through photochemical processes, some molecules such as azobenzene (Azo) [18,19,20,21,22,23,24,25], anthracene [26,27,28,29,30], coumarin [31,32,33,34], and cinnamic acid [35,36,37,38,39] in materials change their structures, leading to partial macroscale deformation. Macroscale deformation can also occur by a photophysical process (photothermal process). The absorbed light can be converted to heat, and the resulting heat is dissipated as the molecules undergo thermal relaxation, thus deforming the materials [40]. In addition, the heat originating from photothermal processes can be utilized to increase the chain mobilities of polymeric materials, resulting in self-healing properties [41,42,43]. Water-responsive actuators can be obtained through a similar principle. Partial swelling or hydration can deform materials at the macroscale. In addition to this simple deformation, functional materials can also respond to multiple stimuli, such as light and moisture. In most cases, multiple stimuli independently or synergistically affect deformation behaviors [44,45,46,47,48,49]. However, a photoresponsive mechanism involving the switching of behavior in the presence of another stimulus (moisture) has been rarely reported. This switching behavior can be an indicator of the surrounding environments when a stimulus is applied to the materials.

Herein, we report photoresponsive materials that can switch their bending directions in response to *W*_c_. A key strategy for the switching behavior was a balance between hydrophilicity and hydrophobicity. To achieve photoresponsiveness, we chose a hydrophobic polymerizable Azo moiety. To select a hydrophilic part, we investigated three different acrylamide-based liquid monomers for the main chain: acryloyl morpholine (ACMO), dimethyl acrylamide (DMAA), and diethyl acrylamide (DEAA). A switching mechanism was incorporated in response to *W*_c_ by copolymerizing acrylamide-based monomers and Azo monomers. The *W*_c_ of the materials significantly affected the deformation (bending) behaviors of the Azo-containing materials. The bending behaviors, molecular scale behaviors, and phase-scale behaviors were investigated by videos, UV–visible (Vis) spectroscopy, and grazing-incidence small-angle X-ray scattering (GISAXS) measurements, respectively. The Azo-containing materials switched their bending directions in response to *W*_c_. This switching behavior can be applied to a UV light sensor, showing the environmental information simultaneously.

## 2. Results and Discussion

As hydrophobicity is a key parameter of interest in this research, we determined the order of the hydrophilicities by comparing the contact angles of the three different liquid monomers and water on a glass substrate. We hypothesized that a contact angle more similar to that of water implies greater hydrophilicity. The contact angles of ACMO, DMAA, and DEAA were approximately 42°, 30°, and 25°, respectively (Figure S1). Hereinafter, we postulated that ACMO was the most hydrophilic and that DEAA was the least hydrophilic.

### 2.1. Preparation of Moisture-Sensitive Photoresponsive Hydrogels

**Figure 1:**
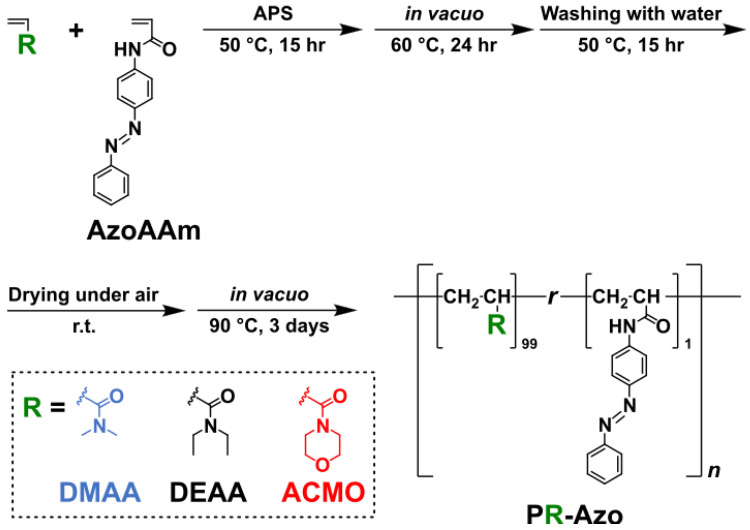
Chemical structures of the moisture-sensitive photoresponsive hydrogels (PDMAA-Azo, PDEAA-Azo, and PACMO-Azo) and details of the polymerization and posttreatment. The hydrogels were prepared in the bulk state. Namely, liquid monomers served as the solvent as well as monomers.

Azo-containing hydrogels were prepared as shown in Figure 1. The Azo monomer (AzoAAm) and initiator (ammonium persulfate, APS) were dissolved in three different acrylamide-type monomers (Tables S1–S3). The mixtures were thermally polymerized to generate polymeric films (PR-Azo). Unreacted monomers and initiators were removed by drying and washing processes. A side reaction involving APS resulted in the formation of chemical cross-links [50], preventing the polymeric films from dissolving.

As it was impossible to dissolve the polymeric film, we analyzed the polymeric films using ^1^H field gradient magic angle spinning (FGMAS) nuclear magnetic resonance (NMR) measurements (Figures S2–S4). By comparing the integral values of the protons on the main chain (*H*_b_) with those of the protons on the Azo moieties (*H*_d_ or *H*_e_), the modification ratios of the Azo moieties were approximately 1 mol%. Subsequently, the macroscale photoresponsive behaviors were investigated using PR-Azo. However, these polymeric films were too thick to investigate molecule-scale photoresponsive behaviors. Moreover, as it was impossible to fabricate extremely thin polymeric films in the manner described above, linear polymers were also prepared to fabricate thin polymeric films.

Thermal polymerization simply resulted in Azo-bearing polymers (Figure S5 and Tables S4–S6). Hereinafter, these polymers are called PR-Azo(sol) because the obtained polymers were soluble in an appropriate solvent. The ^1^H NMR spectra of PR-Azo(sol) showed that the three polymers contained Azo moieties similar to those of PR-Azo (Figures S6–S8). The contents of the Azo moieties were calculated in the same manner as that described above. The three PR-Azo(sol) polymers also contained approximately 1–2 mol% Azo moieties. This similarity implied that we could understand the macroscale behaviors of PR-Azo through molecular-scale studies using PR-Azo(sol).

### 2.2. Photoresponsiveness of Hydrogels in Different Moisture Environments on the Molecular Scale

The molecular-scale behaviors of PR-Azo(sol) were investigated by monitoring changes in the UV–Vis spectra during irradiation with UV or, subsequently, Vis light. To prepare samples for UV–Vis spectroscopy, we fabricated thin polymeric films on a glass substrate using a spin coater (Figure S9 and Table S7). The films were measured in a dry state and a humid state. The humid-state samples were prepared by storing the samples in a container with a wet paper towel at 30 °C for several hours. The polymeric thin films were irradiated by UV light and, subsequently, by Vis light. All irradiations were performed until the UV–Vis spectra reached an equilibrated state.

PACMO-Azo(sol) showed similar photoisomerization behavior toward *cis*-Azo when irradiated with UV light regardless of its water content (Figure S10), while the other isomerization rates differed with water content. The dried PACMO-Azo(sol) required 1600 s to reach the equilibrated state. However, the humid-state sample required only 540 s, implying a three times faster isomerization rate toward *trans*-Azo than that of the dried sample. In the case of PDMAA-Azo(sol), the equilibrated state still had relatively obvious absorbance at approximately 360 nm compared with PACMO-Azo(sol) (Figure S11). Moreover, Vis light irradiation did not result in complete recovery of the original spectra, which was not a result of the slight overlap of the UV–Vis spectra of *trans*-Azo and *cis*-Azo. The remaining peak at approximately 360 nm disappeared when the sample was stored in the humid environment. The isomerization rate of the humid-state sample toward *trans*-Azo was two times faster than that of the dried sample. In the spectrum of PDEAA-Azo(sol), the peak at approximately 360 nm remained more significantly (Figure S12). As shown in the spectrum of PDMAA-Azo(sol), the remaining peak in the spectrum of humid-state PDEAA-Azo(sol) disappeared upon UV light irradiation. As the order of hydrophilicity was DEAA < DMAA < ACMO according to the contact angle measurement, the remaining peak in the spectrum of the dried-state sample seemed to be related to trace water around the Azo molecules.

The time dependence of UV–Vis spectra under UV or Vis light irradiation was plotted based on pseudo-first-order kinetics (Figures S13–S15 and Equation (1)):

(1) $$ln\frac{\left(A_{0}-A_{eq.}\right)}{\left(A_{t}-A_{eq.}\right)}=kt$$

where *A_0_*, *A_t_*, *A_eq._*, *k*, and *t* refer to the initial absorbance, the absorbance at a certain time (*t*), the absorbance at the equilibrated state, a kinetic constant, and the time, respectively. From the slope of the plots, the kinetic constants, *k*_trans→cis_ and *k*_cis→trans_, which refer to the kinetic constants for the isomerizations to *cis*-Azo and *trans*-Azo, respectively, were determined (Table 1 and Table 2). It was found that *k*_cis→trans_ for the humid-state hydrogels significantly increased, while *k*_trans→cis_ did not change that much. These results indicate that water affects the polymeric chains rather than the Azo molecules, because if the Azo molecules were affected *k*_trans→cis_ would also be changed.

### 2.3. Photoresponsiveness of Hydrogels in Different Moisture Environments at the Macro Scale

**Figure 2:**
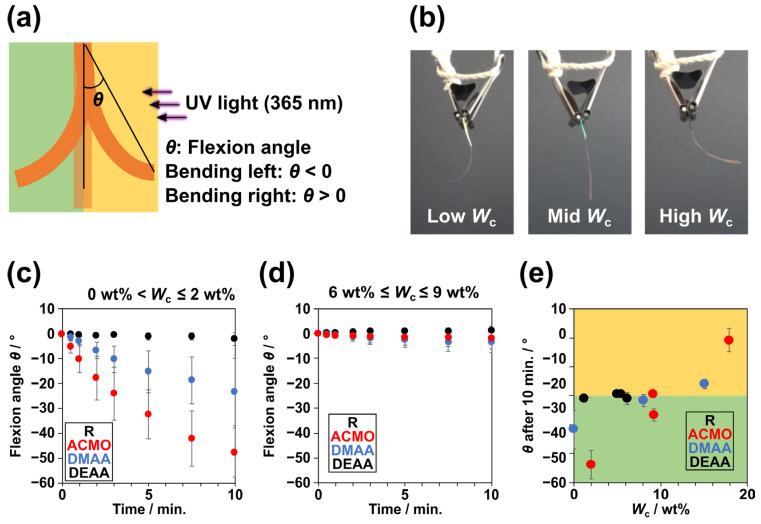
(**a**) Illustration defining the flexion angle *θ* and the positive and negative bending directions. (**b**) Snapshots of PACMO-Azo with three different water contents (*W*_c_) at 8 min. Time dependencies of the flexion angles of PR-Azo with (**c**) low *W*_c_ (0–2 wt%) and (**d**) mid *W*_c_ (6–9 wt%); (**e**) *θ* of PR-Azo after UV light irradiation for 10 min with various *W*_c_ values.

It was revealed that water affected the kinetics of the isomerization of Azo, and the process seems to involve polymeric chains on the molecular scale, as determined from the UV–Vis spectra. The macroscale photoresponsiveness of PR-Azo hydrogels was evaluated on the basis of their bending behaviors under UV light (wavelength 365 nm) irradiation by measuring the flexion angle *θ* (Figure 2a). When a bending occurred toward the light source, it was defined as the positive direction. Namely, bending toward the light source and away from the light source refer to positive and negative *θ* values, respectively. PACMO-Azo with different *W*_c_ values responded differently to UV light (Figure 2b and Videos S1–S3). The *θ* values of PACMO-Azo with low, intermediate (mid), and high *W*_c_ values were negative, approximately 0, and positive, respectively. Subsequently, the time dependencies of *θ* for all PR-Azo samples were investigated (Videos S4–S9). At a low *W*_c_, approximately 0 to 2 wt%, PACMO-Azo and PDMAA-Azo gradually bent away from the light source, while PDEAA-Azo did not bend (Figure 2c). Moreover, PACMO-Azo bent the most. When the polymeric films contained more water, approximately 6 to 9 wt%, all three polymeric samples bent only slightly regardless of the main chain (Figure 2d). This bending usually occurs due to local contraction or expansion of the stimulus-applied area. The switches of the local deformation behaviors with *W*_c_ imply the involvement of a complicated effect of water, not only the photoisomerization of the Azo moieties.

We plotted the *θ* values versus *W*_c_ after 10 min of UV irradiation (Figure 2e). Because of the hydrophobicity of DEAA, the maximum *W*_c_ of PDEAA-Azo was only 6 wt%. The PACMO-Azo and PDMAA-Azo samples switched their bending behaviors at a *W*_c_ value of 10 wt%, which served as a transition point. When the *W*_c_ value was lower than 10 wt%, both PACMO-Azo and PDMAA-Azo bent in the negative direction. The more hydrophilic PACMO-Azo showed a significantly larger *θ* value (approximately −48°) than PDMAA-Azo (approximately −23°). Surprisingly, at a higher *W*_c_ than the transition value, PACMO-Azo and PDMAA-Azo bent in the positive direction. This bending behavior depended on the hydrophilicity of the monomers. More hydrophilic monomers resulted in greater bending. As discussed in the previous section, water molecules seemed to strongly affect bending behaviors.

Although *trans*-Azo required only 1–3 min until it reached the equilibrated states, according to the results obtained from the UV spectroscopies shown in Figures S10–S15, the bending behaviors lasted at least 10 min. These time lags imply an additional mechanism leading to the bending behavior. In general, the relationship between the spatial scale and time scale is proportional. That is, a larger spatial scale requires a longer time. Based on this idea, a discussion from the point of a larger scale should be carried out not only on the molecular scale to deeply understand the bending behaviors.

### 2.4. Structural Studies of Photoresponsive Hydrogels by X-ray Scattering Measurements

In the previous section, the moisture sensitivity of the photoresponsive hydrogels was shown. The change in the bending direction cannot be easily understood by a conventional interpretation based on the molecular level: a collapse of the stacking structure of Azo molecules [21]. To discuss the bending behavior on the phase scale (mesoscale), GISAXS measurements of PACMO-Azo(sol), PDMAA-Azo(sol), and PDEAA-Azo(sol) on a glass substrate were carried out. Following the GISAXS measurement without UV light irradiation, the samples were irradiated entirely with UV light while maintaining the location of the samples to avoid changing the measuring area.

**Figure 3:**
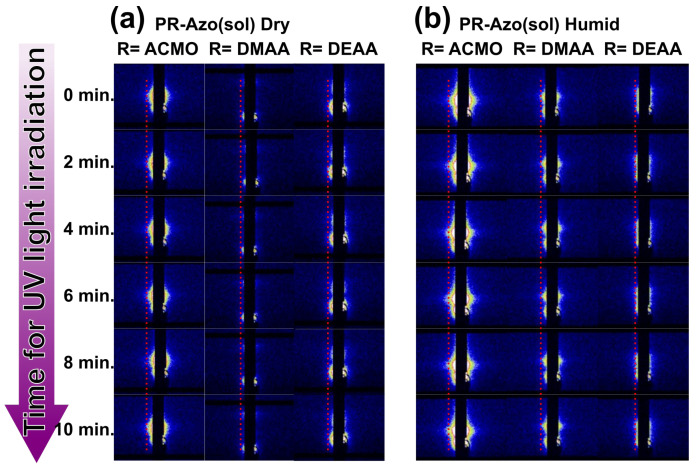
Time dependencies of 2D GISAXS scattering patterns of PR-Azo(sol) in (**a**) the dried states and (**b**) humid states during UV light irradiation.

The dried samples showed relatively small scattering patterns (Figure 3a and Figure S16). Moreover, in the dried states, no sample showed significant changes in the scattering patterns when focusing on the red guide lines, although the UV–Vis spectra changed significantly and bending behaviors (PR-Azo) were observed. The scattering patterns of the X-ray scattering measurement occur due to the fluctuation of electron density. Consequently, no changes in the scattering patterns indicate stable (constant) internal structures on the mesoscale. We postulated that the molecular-scale behavior is more critical than the mesoscale behavior in the dried state, as the bending behaviors away from the light source can be explained by the conventional interpretation.

The humid-state samples showed significantly more scattering patterns than the dried samples (Figure 3b and Figure S17). Interestingly, more hydrophilic polymers showed more significant scattering patterns. These larger scattering patterns imply more heterogeneous structures compared with those in the dried state. We postulated that the larger scattering patterns originated from the domain formation of Azo. As Azo molecules are hydrophobic, they should prefer to aggregate in humid environments. Hence, mesoscale structures of the samples became more heterogenous. As a result, the scattering patterns became more obvious. Upon UV light irradiation, the scattering patterns of PACMO-Azo(sol) increased slightly when focusing on the red guide line in Figure 3. This result can be explained by the aggregation of Azo molecules regardless of the types of isomers, as dictated by the hydrophilic environment. Because both trans-Azo and cis-Azo are hydrophobic, they will form domains in the hydrophilic environment. Moreover, when exposed to the hydrophilic environment, the Azo domain may expand due to the inclusion of a small number of water molecules. For PDMAA-Azo(sol) and PDEAA-Azo(sol), UV light irradiation induced no significant change in the size of the scattering patterns. Less hydrophilicity may not allow the significant domain formation of Azo molecules.

### 2.5. Proposed Bending Mechanism in Response to Water Content

The *W_c_* values were critical to determine the bending direction for PR-Azo. Based on the various investigations described above, we propose bending mechanisms in response to *W_c_*. At low *W_c_* values, the conventional interpretation explains the bending behaviors well. As trans-Azo molecules have a planar structure, they easily form a stacked structure through π-π interactions. Upon irradiation with UV light, the *trans*-Azo molecules isomerize to *cis*-Azo, which has a nonplanar structure, resulting in collapse of the stacked structures. The distance between polymeric chains increases due to the lack of force to bind them close. Therefore, the UV-light-irradiated side expands and bends away from the light source. Despite understanding the bending direction, it was still curious why different *θ* values were observed with different main chains. Most likely, extremely small water molecules around the main chains may help amplify the changes at the molecular scale.

With high *W_c_* values, the driving force for bending toward the light source seems to be hydrophobic interactions. Upon irradiation with UV light, the *trans*-Azo molecules isomerize. Usually, isomerization leads to an increase in the distance between polymeric chains. Here, we suggest that free *cis*-Azo molecules easily aggregate through hydrophobic interactions under certain water contents. The absence of a stacked structure can boost the mobility of the Azo moieties. Consequently, aggregation results in bending behavior toward the light source.

## 3. Conclusions

This report showed the moisture sensitivity of photoresponsive hydrogels with different hydrophilicities. At the molecular scale, *trans*-Azo isomerized to *cis*-Azo upon UV light irradiation regardless of the water content and identity of the main chains, although some differences in the isomerization efficiencies were observed. The most interesting finding was the switching of bending behaviors. At a low *W*_c_ (0–2 wt%), the materials bent away from the light source. On the other hand, the hydrogels bent toward the light source when the *W*_c_ was higher (>10 wt%). The conventional interpretation based on the changes in the distance between polymeric chains only explained the bending behavior at a low *W*_c_. We suggested an aggregation of Azo moieties on a molecular to mesoscale regardless of the isomer types of Azo. In a more hydrophilic matrix, Azo molecules tend to aggregate more due to hydrophobic interactions. Aggregation could result in bending toward the light source. This switching behavior can be applied to sensing materials while simultaneously showing the environmental information of the moment.

Water plays an important role in the bending behaviors of Azo-based hydrogels in this report. The majority of reports focusing on water as a stimulus have usually shown proportional responses (one way). Unlike previous reports, we showed that photoresponsive materials can respond differently according to the *W*_c_. Water did not play the main role, but it significantly changed the actuation behaviors of the materials. We believe that utilizing water as a trigger for switching responsiveness can provide new inspiration for actuating systems in the soft robotics field rather than being used only as a stimulus.

## 4. Materials and Methods

### 4.1. Materials

*N*,*N*-Dimethylacrylamide (DMAA) and CDCl_3_ were purchased from FUJIFILM Wako Pure Chemical Corporation. *N*,*N*-Diethylacrylamide (DEAA) and 4-acrylloylmorpholine (ACMO) were purchased from KJ Chemicals Corporation. Ammonium peroxodisulfate (APS) and 2,2’-azobis(isobutyronitrile) (AIBN) were purchased from Nacalai Tesque Inc. Deuterium oxide (D_2_O) was purchased from Sigma–Aldrich (Louis, MO, USA). The water used for the preparation of the aqueous solutions was purified with a Milli-Q ^®^ Integral MT system. Other reagents were used without further purification. Azobenzene acrylamide (AzoAAm) was prepared according to a previous report [51].

### 4.2. Methods

**Contact angle measurements:** The hydrophilicity of the monomers was determined by contact angle measurements (DMe-211FE, Kyowa Interface Science Co., Ltd., Niiza, Japan) of monomer droplets on cover glasses. A droplet of monomers (5 μL) was placed onto a cover glass via a needle from a syringe. Images of the droplets were captured by a camera and analyzed to obtain the contact angles by fitting the contour of the monomer droplet.

**NMR spectroscopy:** The ^1^H field gradient magic angle spinning (FGMAS) NMR spectra were recorded at 400 MHz with a JEOL JNM-ECA 400 WB NMR spectrometer. The sample spinning rate was 7 kHz with a relaxation delay of 15 *s* at 25 °C. The gel-state samples were swollen with D_2_O or CDCl_3_. The ^1^H NMR spectra of the soluble polymers were recorded with a JEOL ECA-500 NMR spectrometer at 25 °C. The ^13^C NMR spectra of the soluble polymers were recorded with a JEOL ECA-400 NMR spectrometer at 25 °C. Chemical shifts were referenced to solvent values such as HOD (δ = 4.79 ppm for ^1^H) and CDCl_3_ (*δ* = 7.26 ppm for ^1^H and *δ* = 77.16 ppm for ^13^C).

**UV–Vis absorption spectrometer:** The UV–Vis absorption spectra were recorded with a JASCO V-650 at room temperature. In the case of films, measurements were taken in a 1 mm cell. In the case of thin films, measurements were carried out by inserting the entire glass substrate into the site where the light was emitted from the spectrometer. The kinetics of azobenzene moieties was determined according to the Beer–Lambert law and the following equation:

$$\frac{dC}{dt}=-kC$$

where *C*, *t*, and *k* refer to concentration, time, and kinetic constant.

**Photoisomerization:** Azobenzene moieties were isomerized by photoisomerization using a 300 W Xenon lamp (Asahi spectra MAX-301, Tokyo, Japan) equipped with suitable mirror modules (UV mirror module, λ = 250–385 nm; Vis mirror module, λ = 385–740 nm) as a function of the irradiation wavelength. Moreover, to isolate a specific wavelength, a bandpass filter (IAD365) for UV light and a bandpass filter (IADMC450) for visible light were placed on a xenon lamp. The intensities of the transmitted UV light through the bandpass filters (IAD365) using a suitable mirror module were similar to those of Vis light (λ = 450 nm) using the bandpass filter (IADMC450). Film samples (3 mm width × 21 mm length) were fixed on clips and exposed to UV light for 10 min. The distance between the sample and the lamp was fixed at 6 cm. The light irradiation was captured on video, and the bending angle was measured from the video to evaluate the light responsiveness.

**Moisture analysis:** The water contents of the samples were tuned by putting any amount of water and samples in a sealed container and leaving them at 30 °C overnight to absorb water. After all the measurements, the samples were completely dried in vacuo at 90 °C for 3 days. The water contents of the samples (*W*_c_) were calculated by the following equation. *W*_c_ was used as the actual water content of the samples.

*W*_c_ wt % = (*W* − *W*_dry_)/*W* × 100

where *W* is the weight of the sample and *W*_dry_ is the weight of the sample completely dried in vacuo at 90 °C.

**GISAXS measurements:** The 2D GISAXS scattering patterns were obtained from the BL-6A Photon Factory. The wavelength, incident angle of the incident X-ray, and camera length were 1.5 Å, 0.3°, and 1.5 m, respectively. PILATUS3 1M was used as a detector for the measurements. The UV light was irradiated entirely from the top of the samples on the stage without changing the location for 10 min. UV light (wavelength: 365 nm) irradiation was achieved by means of POT-365, Asahi Spectra Inc., Tokyo, Japan.

### 4.3. Preparation of PR-Azo

For PDMAA-Azo as a representative polymer, DMAA (750 µL, 7.3 mmol, 98 equiv.) and AzoAAm (37 mg, 0.15 mmol, 2 equiv.) were added to 5 mL Eppendorf tubes. The monomer solution was purged with nitrogen gas for 15 min, and then APS (8.5 mg, 0.037 mmol, 0.5 equiv.) was added. The solution was stirred to dissolve the initiator, and then it was immediately poured into a Teflon mold (5 cm (width) × 5 cm (length) × 0.5 mm (depth)) and heated at 50 °C for 15 h. After being dried at 60 °C in vacuo for 24 h, the polymer sheets were immersed in excess water (200 times their own weights) for 3 days, exchanging the water twice a day. Then, the swollen hydrogels were dried under air at room temperature, and the four edges were fixed by masking tape to maintain their shape. The amounts of the reagents are summarized in Tables S1–S3.

### 4.4. Preparation of PACMO-Azo(sol)

ACMO (4.4 g, 31 mmol, 98 equiv.), AzoAAm (0.16 g, 0.65 mmol, 2 equiv.), and AIBN (0.0076 g, 0.046 mmol, 0.1 equiv.) were added to a 20 mL PTFE beaker. The solution was purged with nitrogen gas for 15 min. The top of the beaker was covered with Parafilm, and then the solution was heated at 70 °C for 15 h. After the products were dried at 80 °C in vacuo for 24 h, they were dissolved in chloroform (21 mL) and reprecipitated with methanol (300 mL). This reprecipitation process was repeated 3 times. To remove the solvent, the samples were dried at 125 °C in vacuo for 24 h. The amounts of the reagents are summarized in Table S4.

### 4.5. Preparation of PDMAA-Azo(sol)

DMAA (3.9 g, 39 mmol, 98 equiv.), AzoAAm (0.20 g, 0.80 mmol, 2 equiv.), and AIBN (0.0091 g, 0.055 mmol, 0.1 equiv.) were added to a 20 mL PTFE beaker. The solution was purged with nitrogen gas for 15 min. The top of the beaker was covered with Parafilm, and then the solution was heated at 70 °C for 15 h. After being dried at 80 °C in vacuo for 24 h, the samples were dissolved in methanol (30 mL) and reprecipitated with toluene (300 mL). This reprecipitation process was repeated 2 times. To remove residual solvent, they were dried at 125 °C in vacuo for 24 h. The amounts of reagents are summarized in Table S5.

### 4.6. Preparation of PDEAA-Azo(sol)

DEAA (3.7 g, 29 mmol, 98 equiv.), AzoAAm (0.15 g, 0.59 mmol, 2 equiv.), and AIBN (0.0074 g, 0.045 mmol, 0.2 equiv.) were added to a 20 mL PTFE beaker. The solution was purged with nitrogen gas for 15 min. The top of the beaker was covered with Parafilm, and then the sample was heated at 70 °C for 15 h. After the products were dried at 120 °C in vacuo for 2 h, they were dissolved in methanol (20 mL) and dropped into a mixture of methanol (70 mL) and water (210 mL). After being separated from PDEAA-Azo(sol), they were dried at 125 °C in vacuo for 21 h to remove residual solvent. The amounts of the reagents are summarized in Table S6.

## Figures and Tables

**Table 1:** Kinetic constants *k*_trans→cis_ and *k*_cis→trans_ of the photoresponsive hydrogels in the dried state.

R	*k*_trans→cis_/s^−1^	*k*_cis→trans_/s^−1^
ACMO	9.0 ± 3.2 × 10^−3^	3.4 ± 1.2 × 10^−3^
DMAA	1.1 ± 0.66 × 10^−2^	3.6 ± 0.88 × 10^−3^
DEAA	1.3 ± 1.0 × 10^−2^	4.4 ± 1.7 × 10^−3^

**Table 2:** Kinetic constants *k*_trans→cis_ and *k*_cis→trans_ of the photoresponsive hydrogels stored in a humid environment (30 °C, RH = 40%).

R	*k*_trans→cis_/s^−1^	*k*_cis→trans_/s^−1^
ACMO	9.4 ± 2.0 × 10^−3^	6.4 ± 1.3 × 10^−3^
DMAA	1.3 ± 0.30 × 10^−2^	6.1 ± 2.3 × 10^−3^
DEAA	1.9 ± 1.0 × 10^−2^	6.2 ± 2.1 × 10^−3^

## Data Availability

Data are unavailable due to privacy.

## Supplementary Materials

The following supporting information can be downloaded at https://www.mdpi.com/article/10.3390/gels9080658/s1. **Figure S1:** Contact angles of three liquid acrylamide-based monomers and water as a reference on a glass substrate. **Figure S2:** The ^1^H FGMAS NMR spectrum of PACMO-Azo swollen in D_2_O (7 kHz spinning speed, 400 MHz, 25 °C). Based on the integral ratio of the H_b_ and H_d_ peaks, PACMO-Azo contained 1.1% azobenzene. **Figure S3:** The ^1^H FGMAS NMR spectrum of PDMAA-Azo swollen in D_2_O (7 kHz spinning speed, 400 MHz, 25 °C). Based on the integral ratio of the *H*_b_ and *H*_d_ peaks, PDMAA-Azo contained 1.3% azobenzene. **Figure S4:** The ^1^H FGMAS NMR spectrum of PDEAA-Azo swollen in CDCl_3_ (7 kHz spinning speed, 400 MHz, 25 °C). Based on the integral ratio of the *H*_b_ and *H*_d_ peaks, PDEAA-Azo contained 1.1% azobenzene. **Figure S5:** Preparation scheme of water-soluble photoresponsive polymers (PR-Azo(sol)). **Figure S6:** The ^1^H NMR spectrum of water-soluble PACMO-Azo(sol) in CDCl_3_ (500 MHz, 25 °C). The azobenzene content in water-soluble PDEAA-Azo(sol) was 2.0%. **Figure S7:** The ^1^H NMR spectrum of water-soluble PDMAA-Azo(sol) in CDCl_3_ (500 MHz, 25 °C). The azobenzene content in water-soluble PDMAA-Azo(sol) was 1.3%. **Figure S8:** The ^1^H NMR spectrum of water-soluble PDEAA-Azo(sol) in CDCl_3_ (500 MHz, 25 °C). The azobenzene content in water-soluble PDEAA-Azo(sol) was 2.2%. Unreacted AzoAAm and DEAA remained at 12% and 0.4% in PDEAA-Azo(sol), respectively. **Figure S9:** Illustration showing how to prepare a thin film of PR-Azo(sol) on a glass substrate. **Figure S10:** Changes in UV–Vis spectra of PACMO-Azo(sol) under a dry state with (a) UV light irradiation and (b) subsequent Vis light irradiation. Similar changes in UV–Vis spectra of PACNO-Azo(sol) stored in a humid environment with (c) UV light irradiation and (d) subsequent Vis light irradiation. **Figure S11:** Changes in UV–Vis spectra of PDMAA-Azo(sol) under a dry state with (a) UV light irradiation and (b) subsequent Vis light irradiation. Similar changes in UV–Vis spectra of PDMAA-Azo(sol) stored in a humid environment with (c) UV light irradiation and (d) subsequent Vis light irradiation. **Figure S12:** Changes in UV–Vis spectra of PDEAA-Azo(sol) in a dry state with (a) UV light irradiation and (b) subsequent Vis light irradiation. Similar changes in the UV–Vis spectra of PDEAA-Azo(sol) stored in humid environment with (c) UV light irradiation and (d) subsequent Vis light irradiation. **Figure S13:** The pseudo-first-order plots of PACMO-Azo(sol) (a) in the dried state upon UV irradiation, (b) in the dried state upon subsequent Vis light irradiation, (c) in the humid state upon UV irradiation, and (d) in the humid state upon subsequent Vis light irradiation. **Figure S14:** The pseudo-first-order plots of PDMAA-Azo(sol) (a) in the dried state upon UV irradiation, (b) in the dried state upon subsequent Vis light irradiation, (c) in the humid state upon UV irradiation, and (d) in the humid state upon subsequent Vis light irradiation. **Figure S15:** The pseudo-first-order plots of PDEAA-Azo(sol) (a) in the dried state upon UV irradiation, (b) in the dried state upon subsequent Vis light irradiation, (c) in the humid state upon UV irradiation, and (d) in the humid state upon subsequent Vis light irradiation. **Figure S16:** The 2D GISAXS scattering pattern of (a) PACMO-Azo(sol), (b) PDMAA-Azo(sol), and (c) PDEAA-Azo(sol) in the dried state upon UV irradiation for 10 min. **Figure S17:** The 2D GISAXS scattering pattern of (a) PACMO-Azo(sol), (b) PDMAA-Azo(sol), and (c) PDEAA-Azo(sol) in the humid state upon UV irradiation for 10 min. **Table S1:** Amounts of reagents used in the preparation of PACMO-Azo. **Table S2:** Amounts of reagents used in the preparation of PDMAA-Azo. **Table S3:** Amounts of reagents used in the preparation of PDEAA-Azo. **Table S4:** Amounts of reagents used in the preparation of water-soluble PACMO-Azo(sol). **Table S5:** Amounts of reagents used in the preparation of water-soluble PDMAA-Azo(sol). **Table S6:** Amounts of reagents used in the preparation of water-soluble PDEAA-Azo(sol). **Table S7:** Preparation conditions of PR-Azo(sol) thin films. **Video S1:** UV light responsiveness of PACMO-Azo with a low water content. The playback speed was adjusted to ×16. **Video S2:** UV light responsiveness of PACMO-Azo with an intermediate water content. The playback speed was adjusted to ×16. **Video S3:** UV light responsiveness of PACMO-Azo with a high water content. The playback speed was adjusted to ×16. **Video S4:** UV light responsiveness of PDMAA-Azo with a low water content. The playback speed was adjusted to ×16. **Video S5:** UV light responsiveness of PDMAA-Azo with an intermediate water content. The playback speed was adjusted to ×16. **Video S6:** UV light responsiveness of PDMAA-Azo with a high water content. The playback speed was adjusted to ×16. **Video S7:** UV light responsiveness of PDEAA-Azo with a low water content. The playback speed was adjusted to ×16. **Video S8:** UV light responsiveness of PDEAA-Azo with an intermediate water content. The playback speed was adjusted to ×16. **Video S9:** UV light responsiveness of PDEAA-Azo with a high water content. The playback speed was adjusted to ×16.
