# Supplementary material for: Water-Content-Dependent Switching of the Bending Behavior of Photoresponsive Hydrogels Composed of Hydrophilic Acrylamide-Based Main Chains and Hydrophobic Azobenzene
Source: Gels. 2023 Aug 16;9(8):658. doi: 10.3390/gels9080658 (PMC10453818; doi:10.3390/gels9080658)
Supplement: Supplementary file 1 [file gels-09-00658-s001.zip › gels-2476398_SI_230807.pdf]

-Supplementary Materials-

Water Content-Dependent Switching of the Bending Behavior of Photoresponsive Hydrogels Composed of Hydrophilic Acrylamide-Based Main Chains and Hydrophobic Azobenzene

Junsu Park [1,3], Yuki Shimizu [1], Xin Zhou [1], Ryohei Ikura [1,3],*
*Go Matsuba [5], and Yoshinori Takashima [1,2,3,4]**

1. Department of Macromolecular Science, Graduate School of Science, Osaka University, 1-1 Machikaneyama, Toyonaka, Osaka 560-0043, Japan.
2. Institute for Advanced Co-Creation Studies, Osaka University, 1-1 Yamadaoka, Suita, Osaka, 565-0871, Japan.
3. Forefront Research Center, Graduate School of Science, Osaka University, 1-1 Machikaneyama, Toyonaka, Osaka, 560-0043, Japan.
4. Innovative Catalysis Science Division, Institute for Open and Transdisciplinary Research Initiatives (OTRI), Osaka University, 1-1 Yamadaoka, Suita, Osaka 565-0871, Japan.
5. Graduate School of Organic Materials Engineering, Yamagata University, 4-3-16 Jonan, Yonezawa, Yamagata 992-8510, Japan.

E-mail: parkj18@chem.sci.osaka-u.ac.jp, takasima@chem.sci.osaka-u.ac.jp

Contents

1. Contact angles of acrylamide-type monomers and water on a glass substrate 3

2. Preparation of photoresponsive hydrogels (PR-Azo) 4

3. FGMAS NMR spectra of PR-Azo hydrogels 5

4. Preparation of water-soluble photoresponsive polymers (PR-Azo(sol)) 7

5. NMR spectra of water-soluble PR-Azo(sol) 8

6. Preparation of a thin film on a glass substrate by spin coating 10

7. Changes in UV–Vis spectra of PR-Azo(sol) during light irradiation in dry and humid environments 11

8. Kinetic studies of azobenzene in PR-Azo(sol) during light irradiation in dry and humid environments 14

9. 2D GISAXS scattering patterns of PR-Azo(sol) during UV light irradiation in dry and humid environments 17

1. Contact angles of acrylamide-type monomers and water on a glass substrate

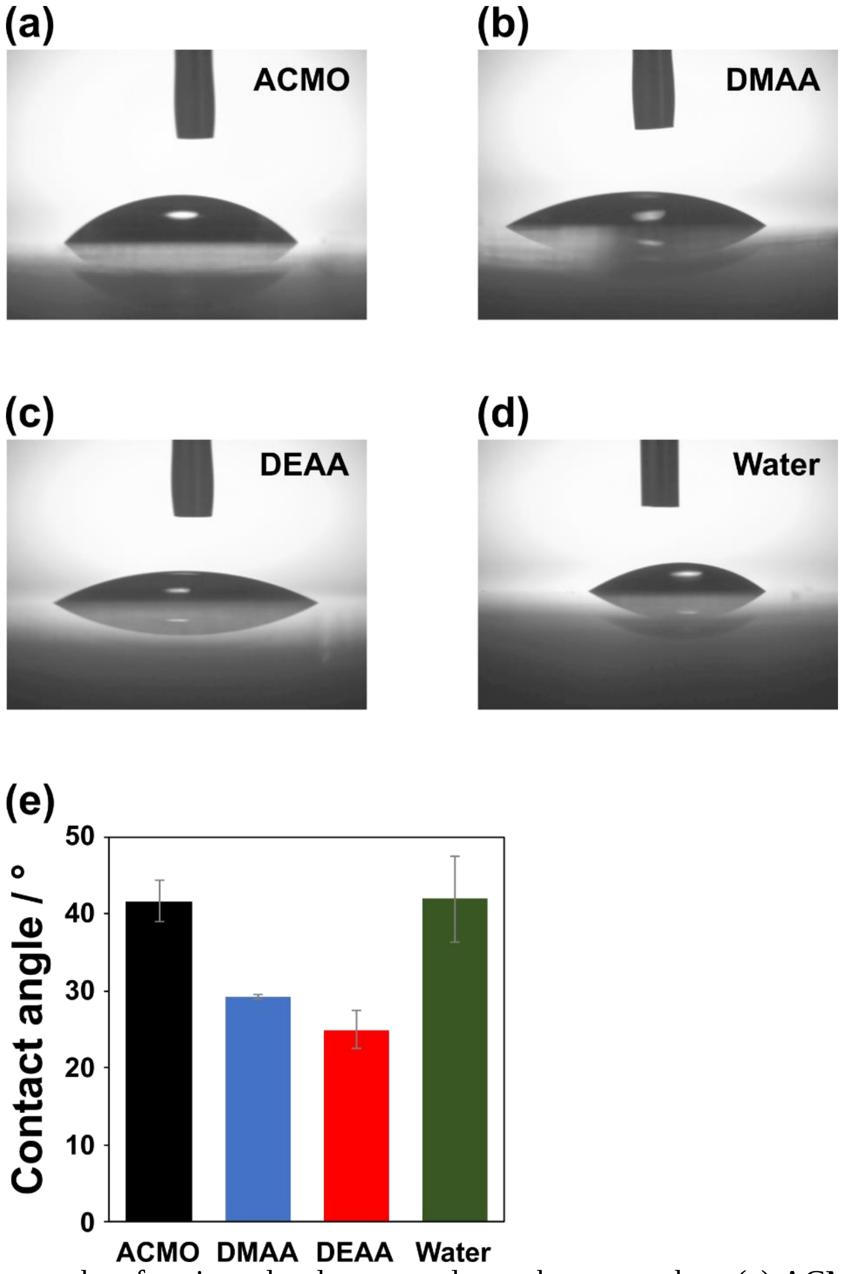

Figure S1. Photographs of various droplets on a glass substrate such as (a) ACMO, (b) DMAA, (c) DEAA, and (d) water. (e) Contact angles of three liquid acrylamide-based monomers and water as a reference on a glass substrate.

2. Preparation of photoresponsive hydrogels (PR-Azo)

Table S1. Amounts of reagents used in the preparation of PACMO-Azo.

ACMO / μL (mmol)	AzoAAm / mg (mmol)	APS / mg (mmol)
750 (6.0)	31 (0.12)	6.9 (0.030)

Table S2. Amounts of reagents used in the preparation of PDMAA-Azo.

DMAA / μL (mmol)	AzoAAm / mg (mmol)	APS / mg (mmol)
750 (7.3)	37 (0.15)	8.5 (0.037)

Table S3. Amounts of reagents used in the preparation of PDEAA-Azo.

DEAA / μL (mmol)	AzoAAm / mg (mmol)	APS / mg (mmol)
750 (5.4)	28 (0.11)	6.3 (0.028)

3. FGMAS NMR spectra of PR-Azo hydrogels

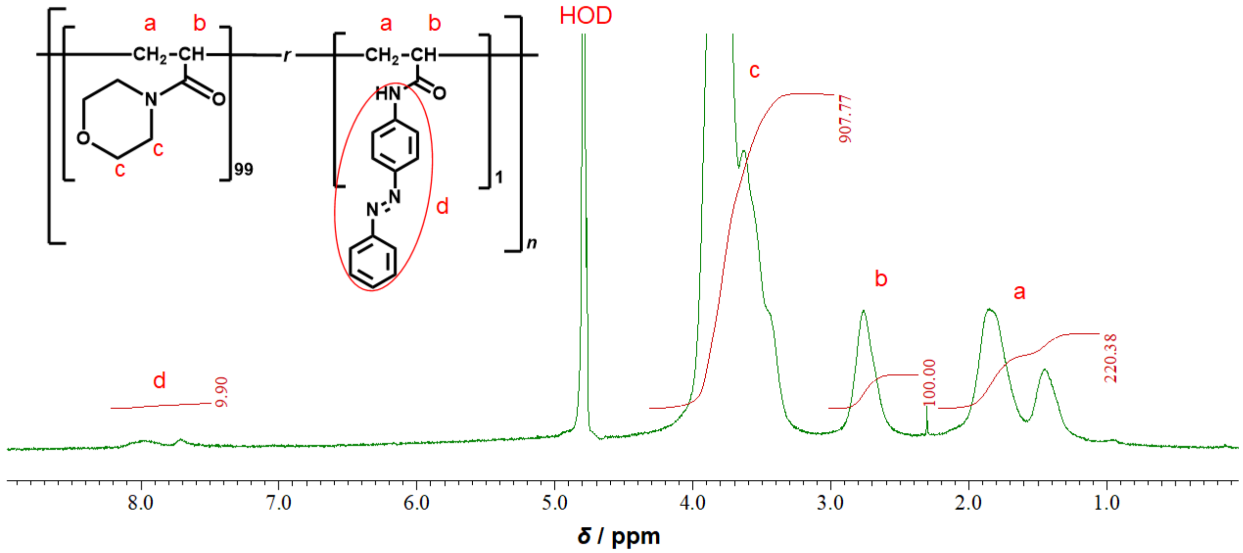

Figure S2. ^{1}H FGMAS NMR spectrum of PACMO-Azo swollen in D_2O (7 kHz spinning speed, 400 MHz, 25 °C). Based on the integral ratio of the H_b and H_d peaks, PACMO-Azo contained 1.1% azobenzene.

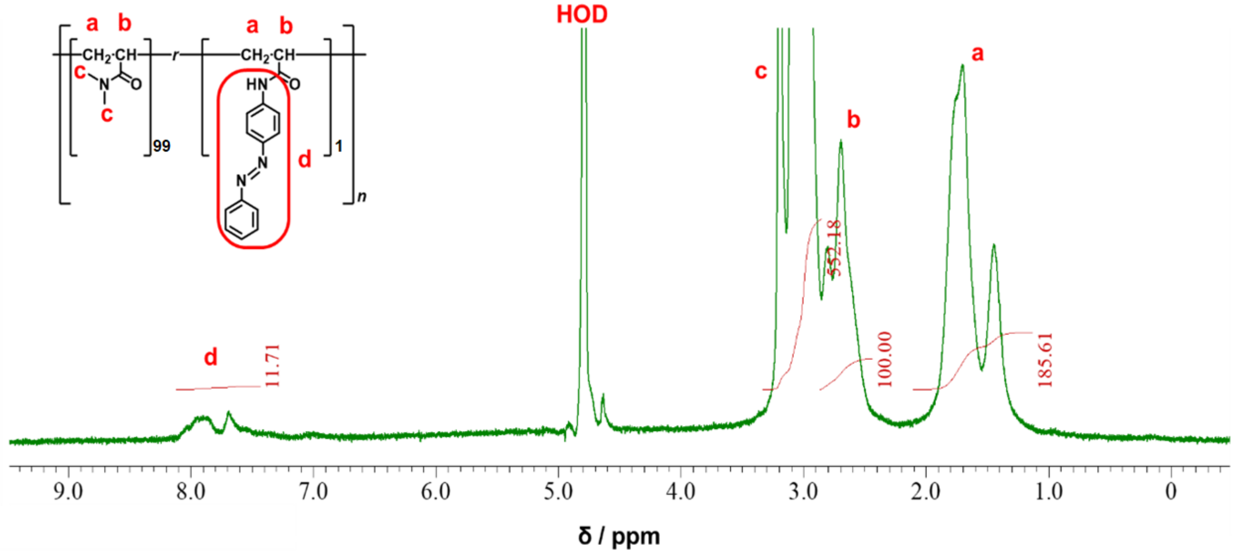

Figure S3. ^{1}H FGMAS NMR spectrum of PDMAA-Azo swollen in D_2O (7 kHz spinning speed, 400 MHz, 25 °C). Based on the integral ratio of the H_b and H_e peaks, PDMAA-Azo contained 1.3% azobenzene.

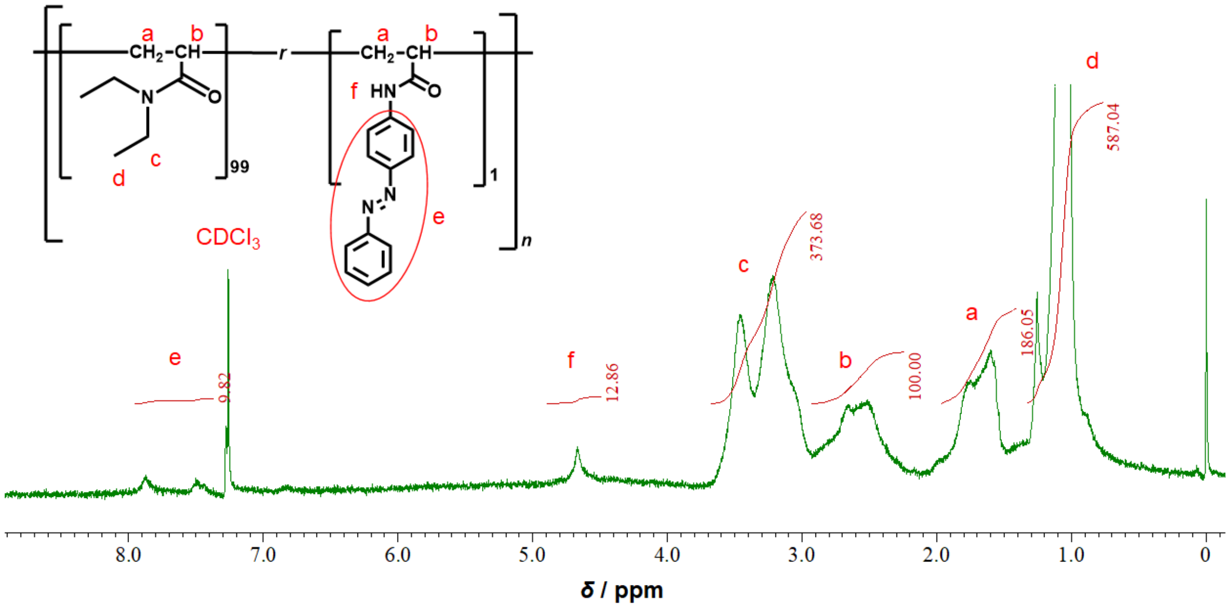

Figure S4. ^{1}H FGMAS NMR spectrum of PDEAA-Azo swollen in $CDCl_3$ (7 kHz spinning speed, 400 MHz, 25 °C). Based on the integral ratio of the H_b and H_d peaks, PDEAA-Azo contained 1.2% azobenzene.

4. Preparation of water-soluble photoresponsive polymers (PR-Azo(sol))

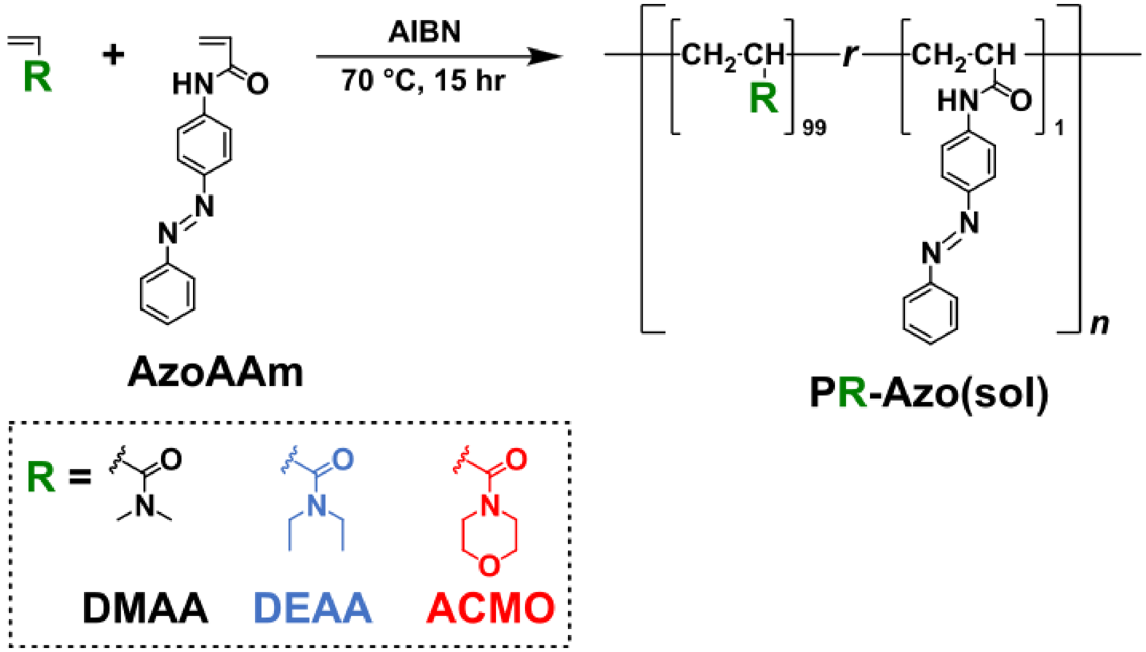

Figure S5. Preparation scheme of water-soluble photorespoinve polymers (PR-Azo(sol)).

Table S4. Amounts of reagents used in the preparation of water-soluble PACMO-Azo(sol).

ACMO / mg (mmol)	AzoAAm / mg (mmol)	AIBN / mg (mmol)
4400 (31)	160 (0.65)	7.6 (0.046)

Table S5. Amounts of reagents used in the preparation of water-soluble PDMAA-Azo(sol).

DMAA / mg (mmol)	AzoAAm / mg (mmol)	AIBN / mg (mmol)
3900 (39)	200 (0.80)	9.1 (0.055)

Table S6. Amounts of reagents used in the preparation of water-soluble PDEAA-Azo(sol).

DEAA / mg (mmol)	AzoAAm / mg (mmol)	AIBN / mg (mmol)
3700 (29)	150 (0.59)	7.4 (0.045)

5. NMR spectra of water-soluble PR-Azo(sol)

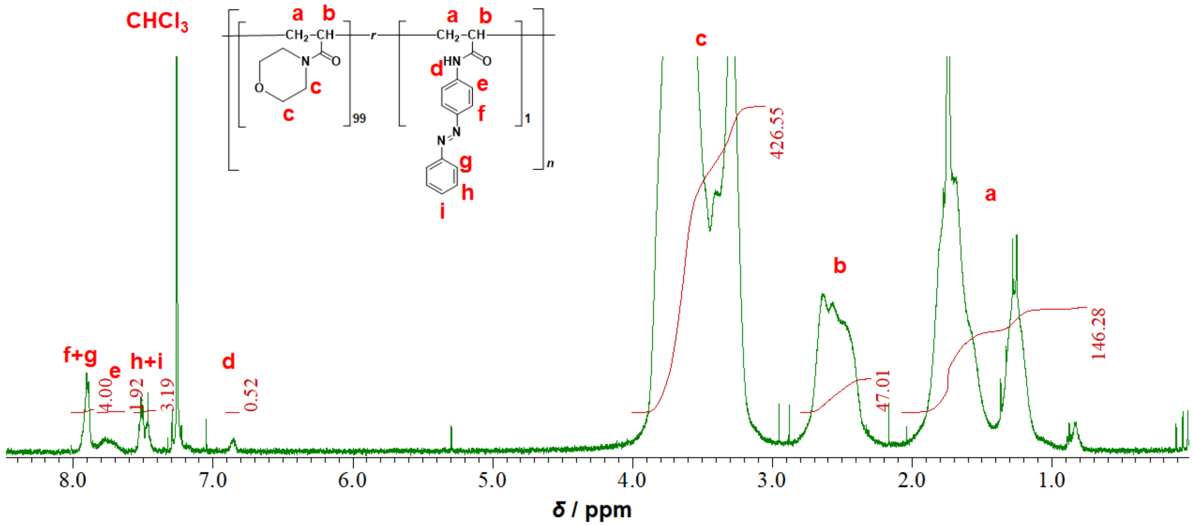

Figure S6. ¹H NMR spectrum of water-soluble PACMO-Azo(sol) in $CDCl_3$ (500 MHz, 25 °C). Based on the integral ratio of the H_b and H_{f+g} peaks, the azobenzene content in water-soluble PACMO-Azo(sol) was 2.1%.

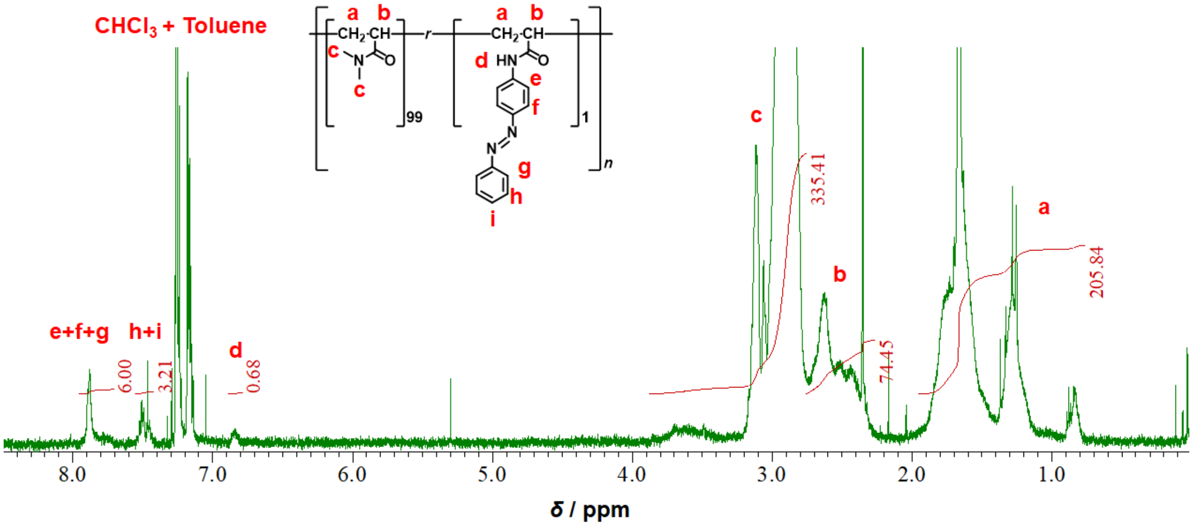

Figure S7. ¹H NMR spectrum of water-soluble PDMAA-Azo(sol) in $CDCl_3$ (500 MHz, 25 °C). Based on the integral ratio of the H_b and H_{e+f+g} peaks, the azobenzene content in water-soluble PDMAA-Azo(sol) was 1.3%.

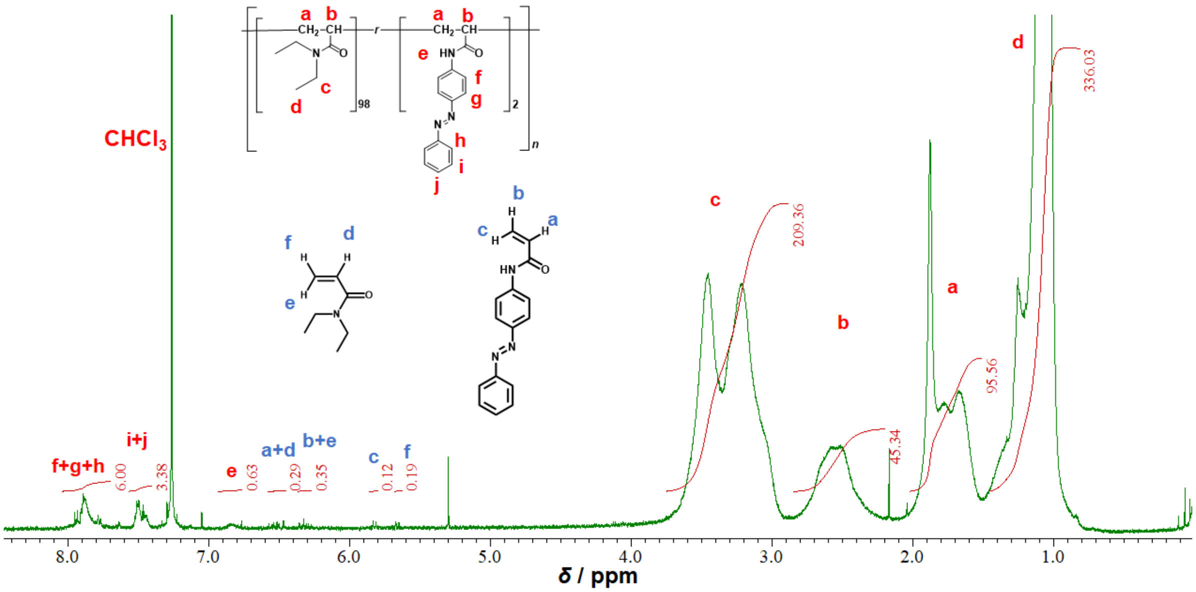

Figure S8. ^{1}H NMR spectrum of water-soluble PDEAA-Azo(sol) in $CDCl_3$ (500 MHz, 25 °C). Based on the integral ratio of the H_b and H_{f+g} peaks, The azobenzene content in water-soluble PDEAA-Azo(sol) was 2.2%. Unreacted AzoAAm and DEAA remained at concentrations of 12% and 0.4% in PDEAA-Azo(sol), respectively.

6. Preparation of a thin film on a glass substrate by spin coating

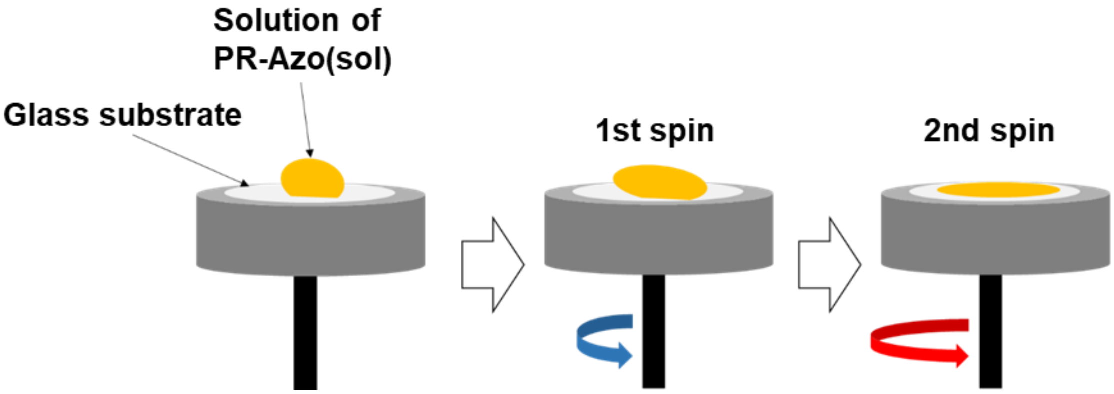

Figure S9. Illustration showing how to prepare a thin film of PR-Azo(sol) on a glass substrate.

Table S7. Preparation conditions of PR-Azo(sol) thin films.

R	Solution concentrations / wt%	Rotation conditions
**DMAA**	8	1st spin: 4000 rpm for 5 s<br>2nd spin: 8000 rpm for 15 s
**DEAA**	8	1st spin: 2000 rpm for 5 s<br>2nd spin: 8000 rpm for 15 s
**ACMO**	6	1st spin: 4000 rpm for 5 s<br>2nd spin: 8000 rpm for 15 s

7. Changes in UV–Vis spectra of PR-Azo(sol) during light irradiation in dry and humid environments

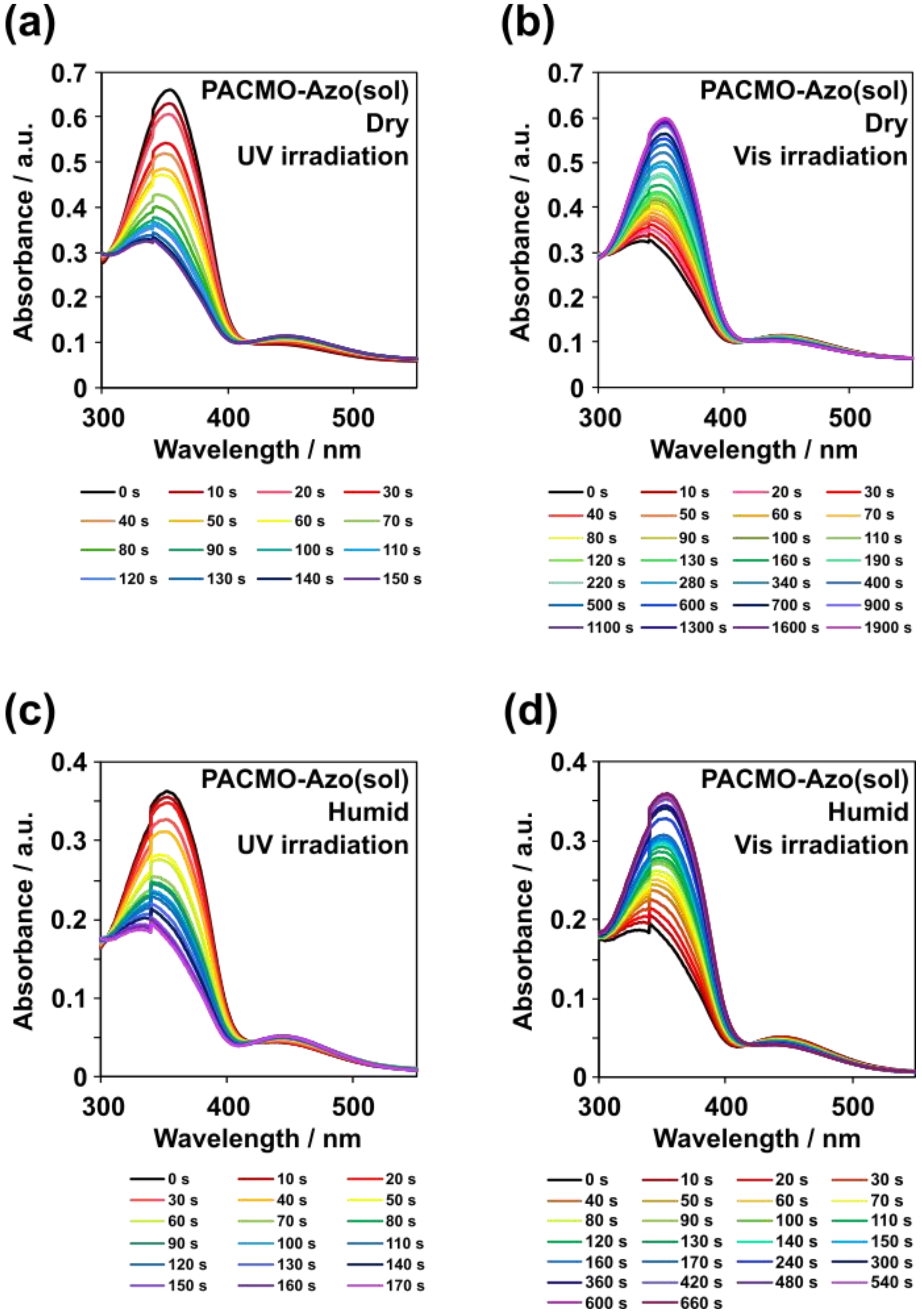

Figure S10. Changes in the UV–Vis spectra of PACMO-Azo(sol) in a dried state upon (a) UV light irradiation and (b) subsequent Vis light irradiation. Similar changes in the UV–Vis spectra of PACMO-Azo(sol) stored in a humid environment upon (c) UV light irradiation and (d) subsequent Vis light irradiation.

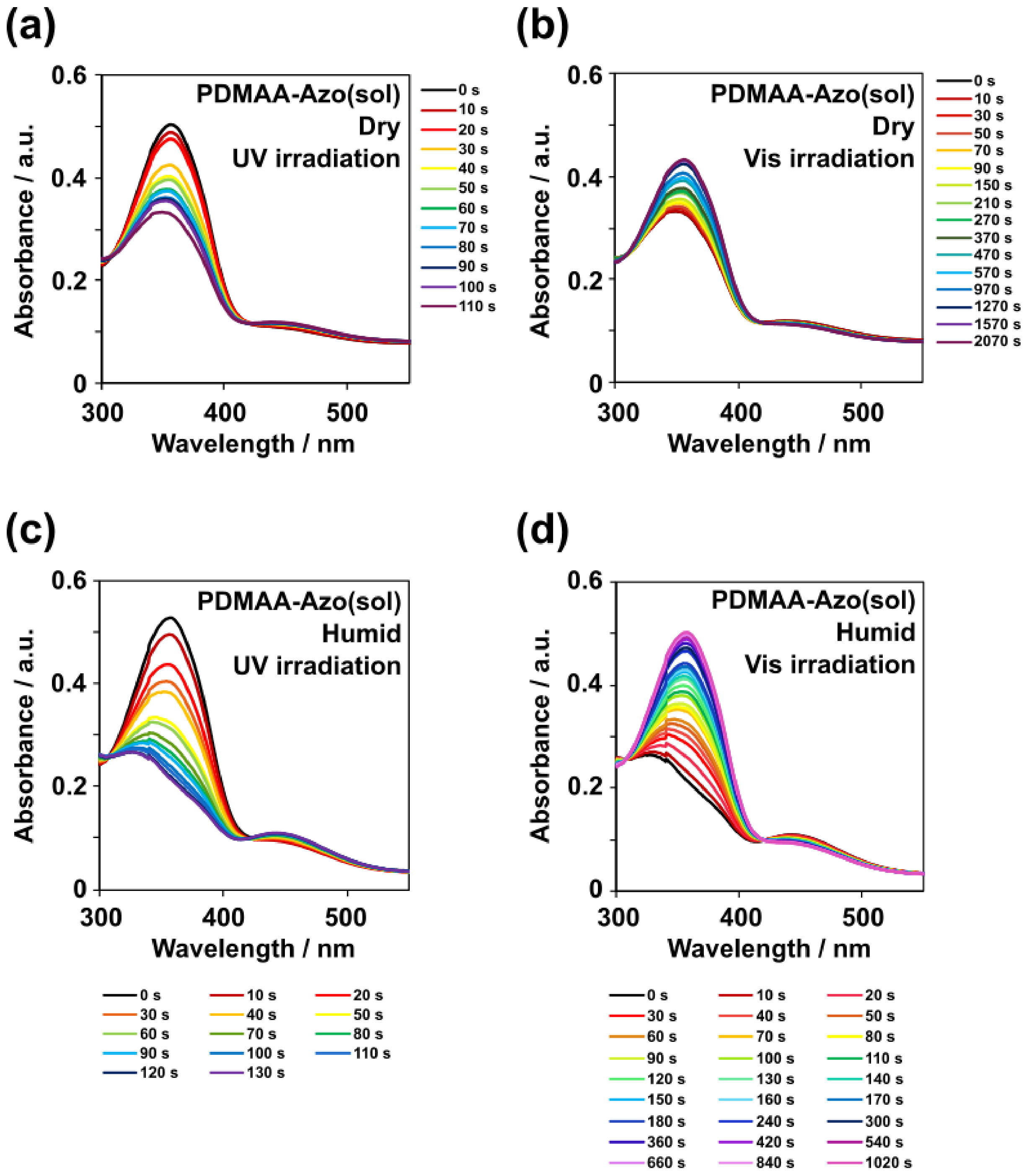

Figure S11. Changes in the UV–Vis spectra of PDMAA-Azo(sol) in a dried state upon (a) UV light irradiation and (b) subsequent Vis light irradiation. Similar changes in the UV–Vis spectra of PDMAA-Azo(sol) stored in a humid environment upon (c) UV light irradiation and (d) subsequent Vis light irradiation.

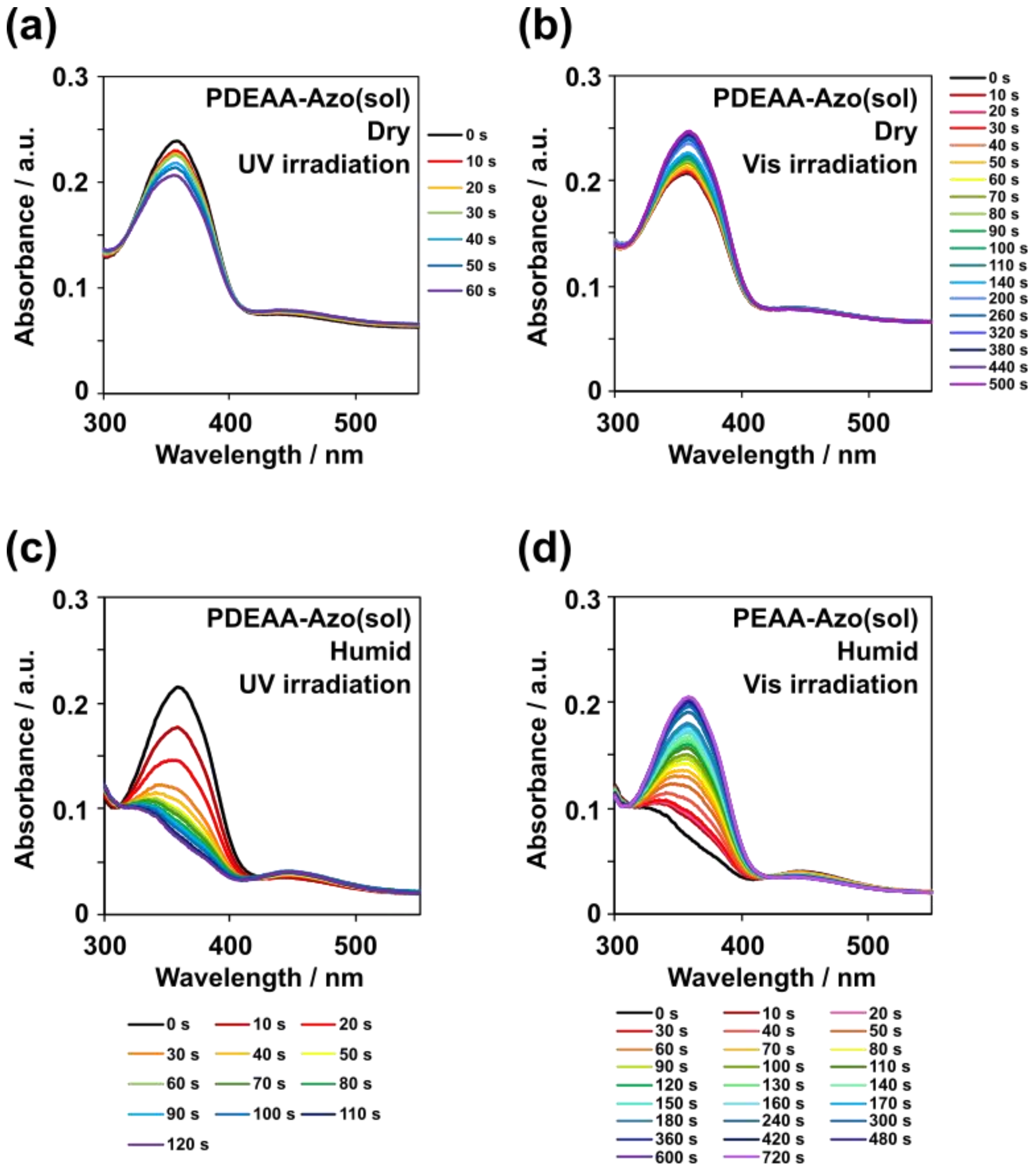

Figure S12. Changes in the UV–Vis spectra of PDEAA-Azo(sol) in the dried state uon (a) UV light irradiation and (b) subsequent Vis light irradiation. Similar changes in the UV–Vis spectra of PDEAA-Azo(sol) stored in a humid environment upon (c) UV light irradiation and (d) subsequent Vis light irradiation.

8. Kinetic studies of azobenzene in PR-Azo(sol) during light irradiation in dry and humid environments

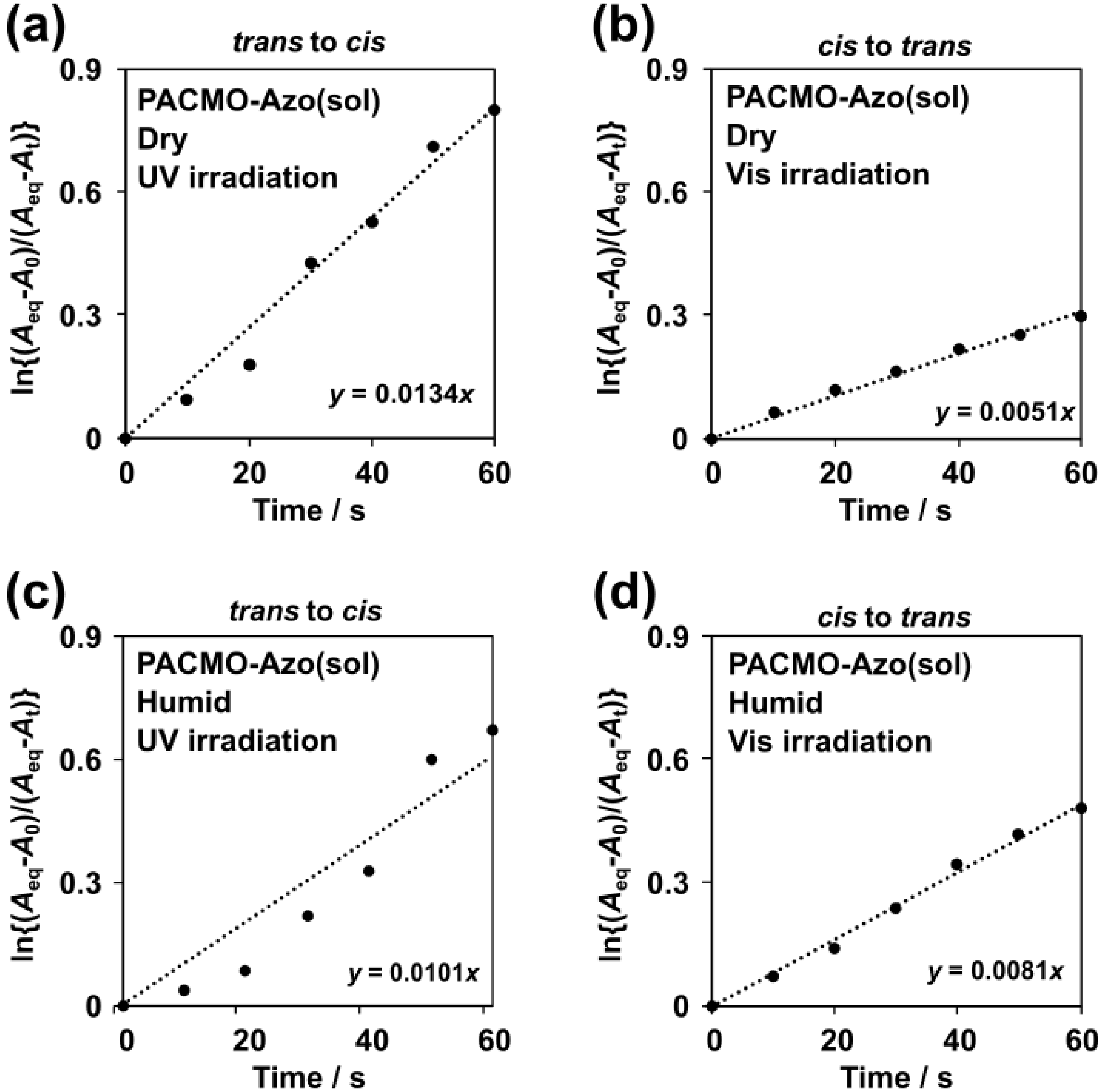

Figure S13. Pseudo-first-order plots for PACMO-Azo(sol) (a) in the dry state upon UV irradiation, (b) in the dry state upon subsequent Vis light irradiation, (c) in the humid state upon UV irradiation, and (d) in the humid state upon subsequent Vis light irradiation

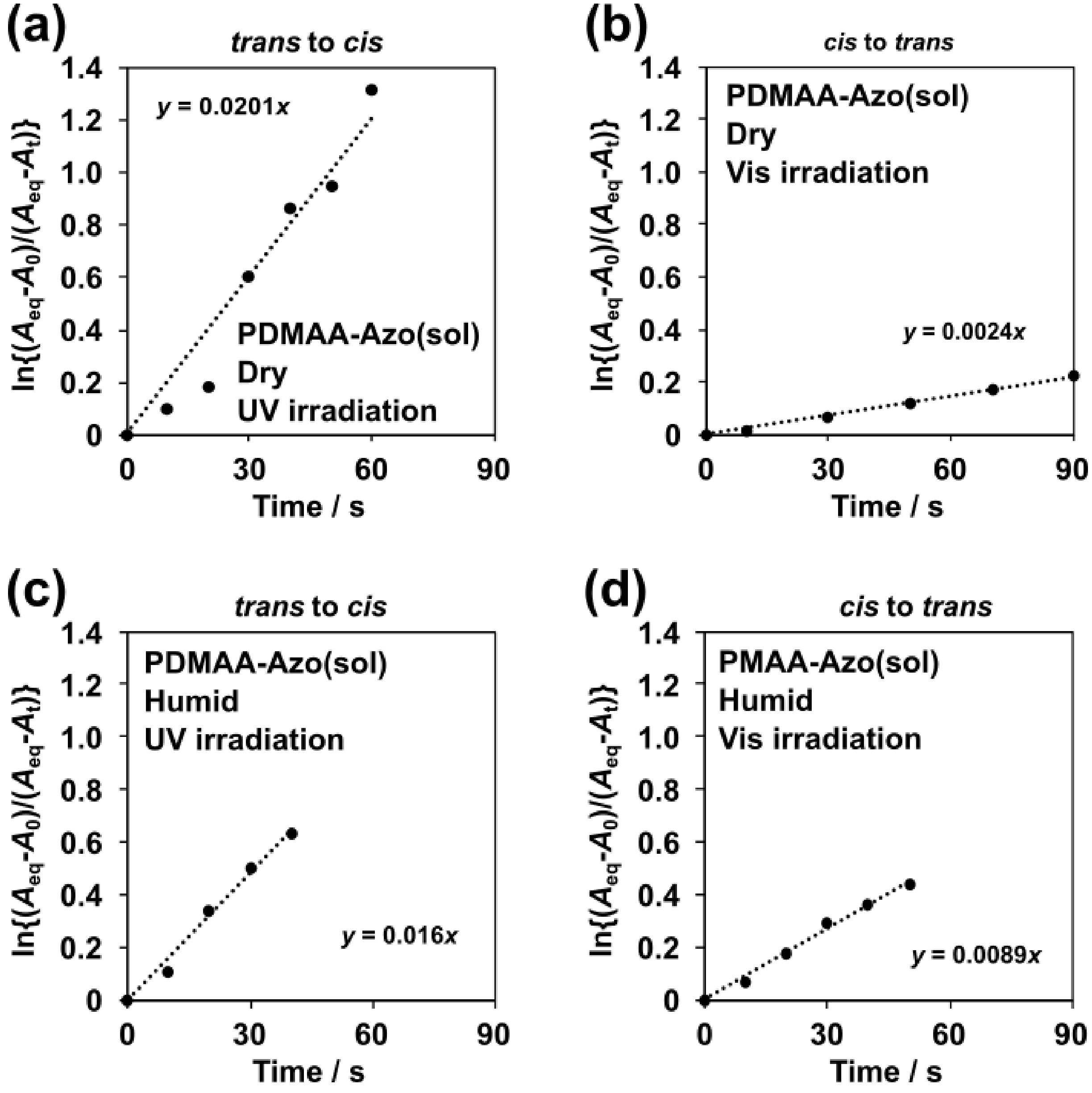

Figure S14. Pseudo-first-order plots of PDMAA-Azo(sol) (a) in the dry state upon UV irradiation, (b) in the dry state upon subsequent Vis light irradiation, (c) in the humid state upon UV irradiation, and (d) in the humid state upon subsequent Vis light irradiation.

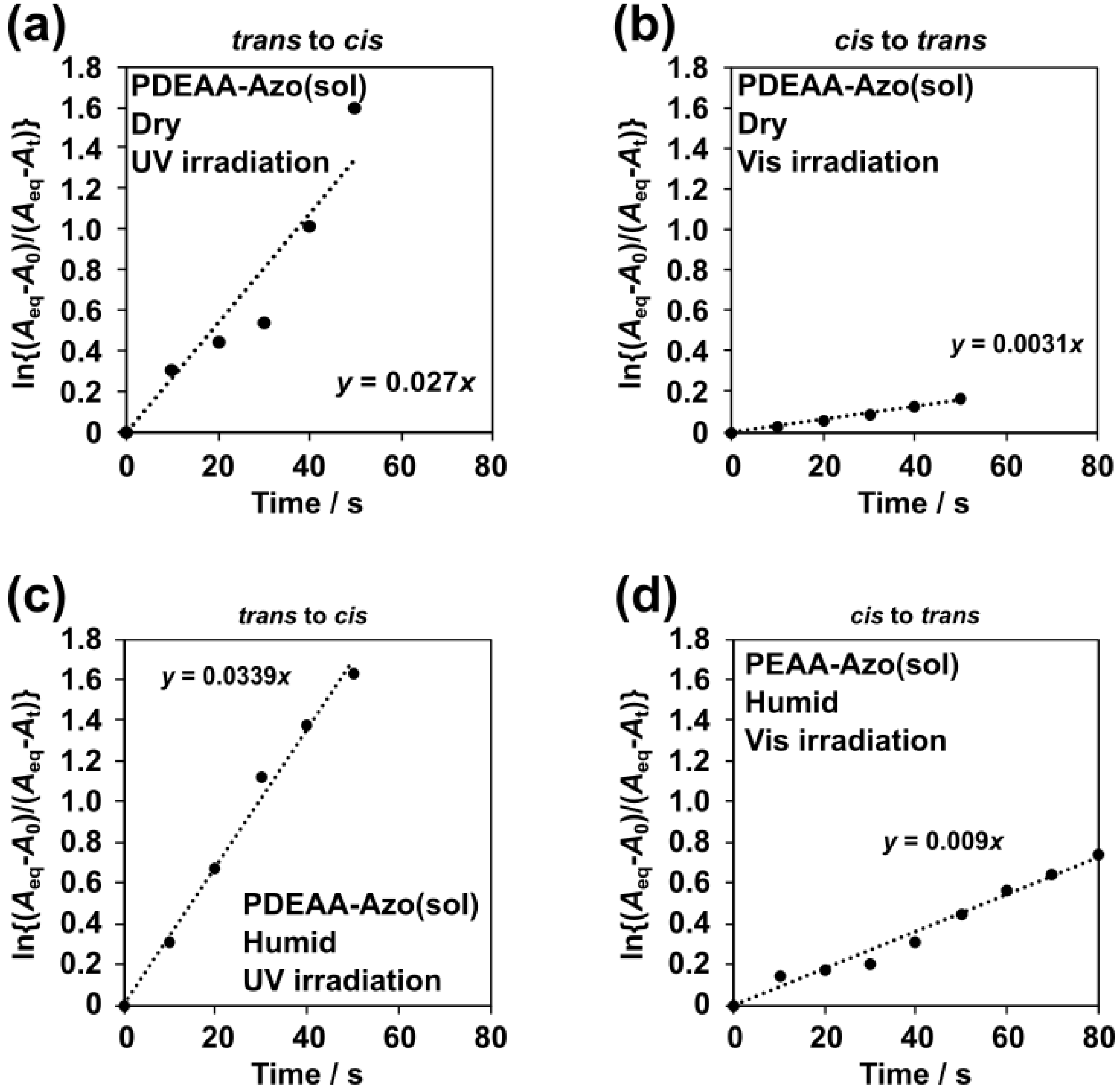

Figure S15. Pseudo-first-order plots of PDEAA-Azo(sol) (a) in the dry state upon UV irradiation, (b) in the dry state upon subsequent Vis light irradiation, (c) in the humid state upon UV irradiation, and (d) in the humid state upon subsequent Vis light irradiation.

9. 2D GISAXS scattering patterns of PR-Azo(sol) during UV light irradiation in dry and humid environments

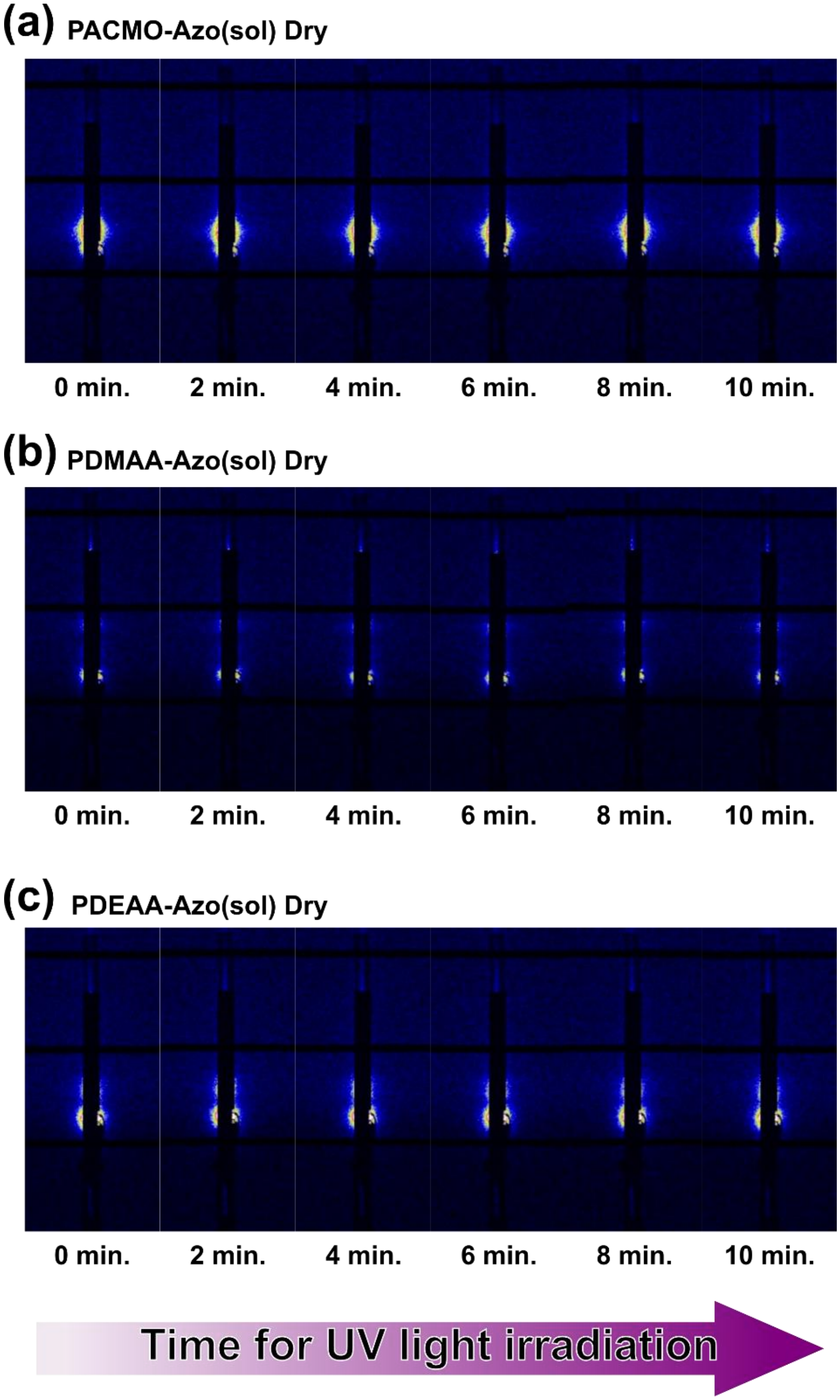

Figure S16. 2D GISAXS scattering patterns of (a) PACMO-Azo(sol), (b) PDMAA-Azo(sol), and (c) PDEAA-Azo(sol) in the dry state upon UV irradiation for 10 minutes.

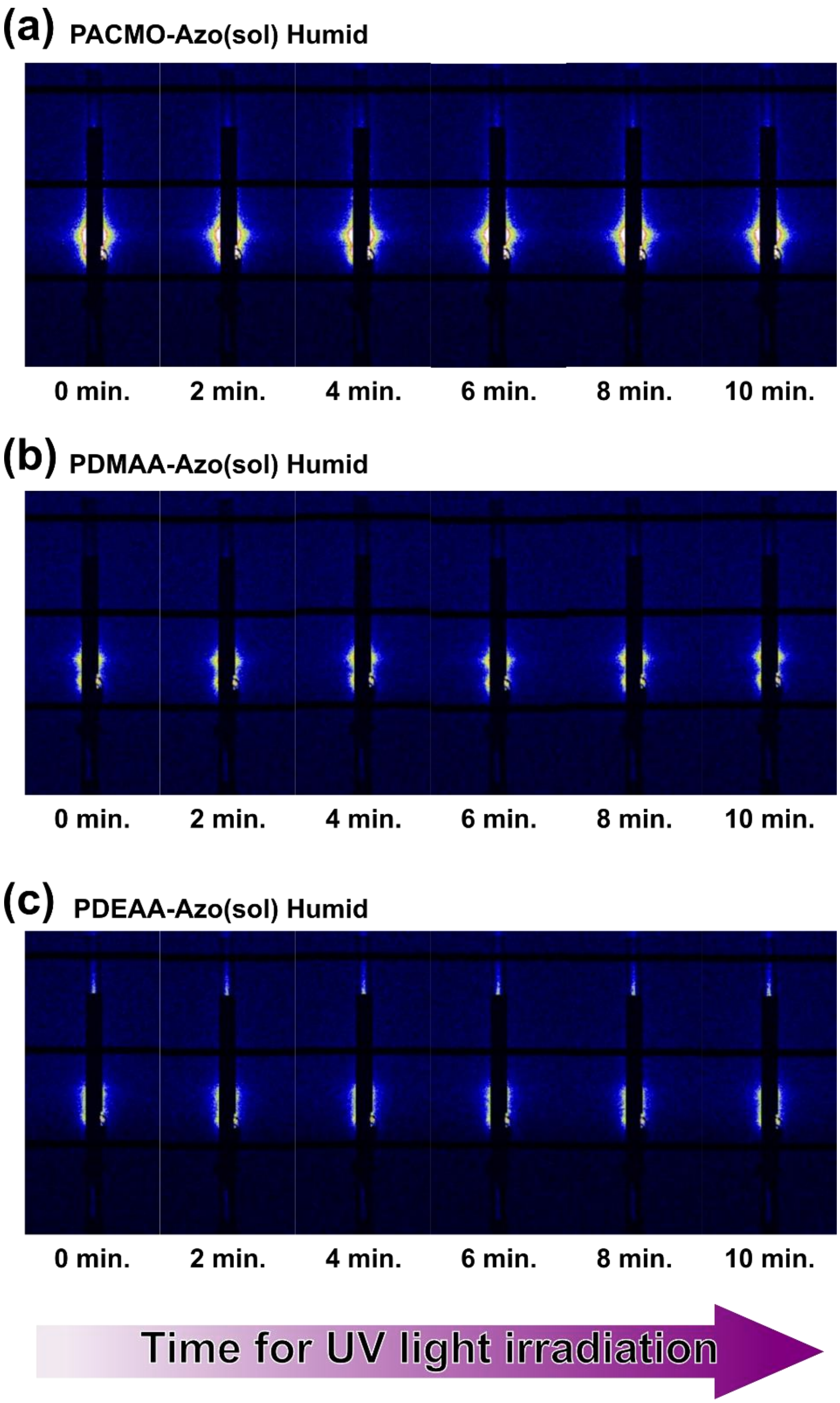

Figure S17. 2D GISAXS scattering patterns of (a) PACMO-Azo(sol), (b) PDMAA-Azo(sol), and (c) PDEAA-Azo(sol) in the humid state upon UV irradiation for 10 minutes.
